# Oscillations in well-mixed, deterministic feedback systems: Beyond ring oscillators

**DOI:** 10.1016/j.jtbi.2019.05.004

**Published:** 2019-11-21

**Authors:** Karen M. Page

**Affiliations:** Department of Mathematics, University College London, Gower Street, London WC1E 6BT, U.K.

**Keywords:** Oscillations, Hopf bifurcation, Gene regulatory network, Network motif, Ring oscillator

## Abstract

A ring oscillator is a system in which one species regulates the next, which regulates the next and so on until the last species regulates the first. In addition, the number of the regulations which are negative, and so result in a reduction in the regulated species, is odd, making the overall feedback in the loop negative. In ring oscillators, the probability of oscillations is maximised if the degradation rates of the species are equal. When there is more than one loop in the regulatory network, the dynamics can be more complicated. Here, a systematic way of organising the characteristic equation of ODE models of regulatory networks is provided. This facilitates the identification of Hopf bifurcations. It is shown that the probability of oscillations in non-ring systems is maximised for unequal degradation rates. For example, when there is a ring and a second ring employing a subset of the genes in the first ring, then the probability of oscillations is maximised when the species in the sub-ring degrade more slowly than those outside, for a negative feedback subring. When the sub-ring forms a positive feedback loop, the optimal degradation rates are larger for the species in the sub-ring, provided the positive feedback is not too strong. By contrast, optimal degradation rates are smaller for the species in the sub-ring, when the positive feedback is very strong. Adding a positive feedback loop to a repressilator increases the probability of oscillations, provided the positive feedback is not too strong, whereas adding a negative feedback loop decreases the probability of oscillations. The work is illustrated with numerical simulations of example systems: an autoregulatory gene model in which transcription is downregulated by the protein dimer and three-species and four-species gene regulatory network examples.

## Highlights

• I present a way of breaking down regulatory networks to find Hopf bifurcations. This helps find optimal conditions for oscillations in dynamical systems models of these networks.
• In a model of negative auto-regulation of a gene by its dimeric protein, it is optimal for the monomer to degrade faster than the mRNA and the mRNA to degrade faster than the dimer.
• Adding a weak positive feedback loop to a repressilator increases the probability of oscillations.
• The optimal degradation rate of species in the sub-loop is higher than that of species outside it.
• The opposite is true for a negative feedback sub-loop or a very strong positive feedback sub-loop.

## Graphical abstract

**Figure:**
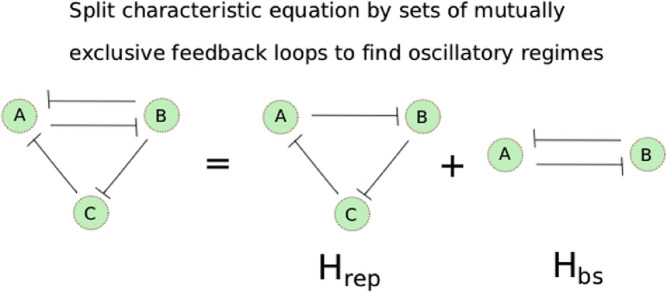

## 1 Introduction

Oscillations in gene expression are important in a variety of biological processes, from circaidian rhythms (Reddy and Rey, 2014) to somitogenesis (Dequéant, Glynn, Gaudenz, Wahl, Chen, Mushegian, Pourquié, 2006, Hirata, Yoshiura, Ohtsuka, Bessho, Harada, Yoshikawa, Kageyama, 2002) to apoptosis in response to DNA damage (Bar-Or, Maya, Segel, Alon, Levine, Oren, 2000, Michael, Oren, 2003). Determining the conditions necessary for oscillations to occur is thus important in the understanding of these processes.

**Fig. 1:**
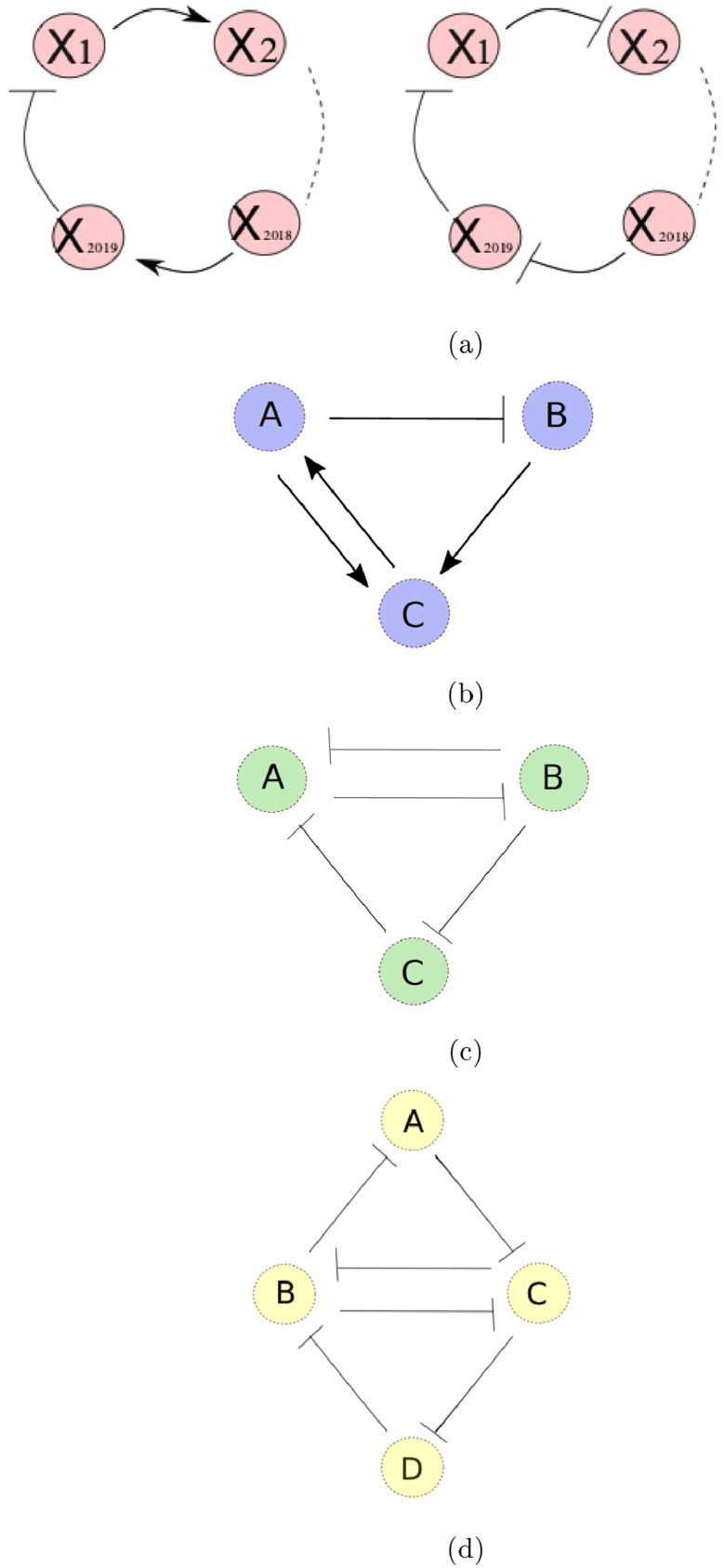
(a) Diagrams of ring oscillators. The biochemical species indicated by $X_{1},X_{2},\cdots ,X_{2019}$ regulate each other in a cyclic manner. There is an odd number of repressions. The diagram on the right hand side depicts a repressilator. (b) Diagram of species interactions in the autoregulatory gene model with dimeric transcription factor. *A* is the protein dimer, *B* the mRNA and *C* the protein monomer. (c) Diagram of the ACDC motif. (d) Diagram of the four species network considered in Section 4.

A ring oscillator is typically a gene regulatory network, containing a single negative feedback loop between the biochemical species. Gene/ biochemical species 1 regulates the expression of species 2 which regulates species 3 and so on. The final species (species *n*) regulates species 1, see Fig. 1a. A species can up-regulate or down-regulate the next. Up-regulations are indicated by sharp-headed arrows and down-regulations by flat-headed arrows, see Alon (2007) for a review of network motifs and De Jong (2002) for a review of mathematical models of gene regulatory networks. If the number of down-regulations in the cycle is odd, so that the feedback loop for species 1 (or any other species) on itself is negative, oscillations are expected (Smith, 1987, Müller, Hofbauer, Endler, Flamm, Widder, Schuster, 2006, Buşe, Kuznetsov, Pérez, 2009, Buşe, Pérez, Kuznetsov, 2010). If the number of down-regulations is even, so that the feedback loop is positive, multi-stability is expected (Smith, 1987, Plahte, Mestl, Omholt, 1995, Cinquin, Demongeot, 2002, Soulé, 2003, Angeli, Ferrell, Sontag, 2004, Müller, Hofbauer, Endler, Flamm, Widder, Schuster, 2006, Kaufman, Soulé, 2019), although long-lived oscillations are possible, for example, in systems with stochasticity (Strelkowa and Barahona, 2010).

Previous work has shown that when the systems giving rise to oscillations are ring oscillators, that is they contain a single negative feedback loop between the chemical species, oscillations are most likely if the degradation rates of all species are equal (Page and Perez Carrasco, 2018). Frequently, oscillations arise in regulatory systems which are more complicated and contain more than one feedback loop, see for example (Panovska-Griffiths, Page, Briscoe, 2013, Perez Carrasco, Barnes, Schaerli, Isalan, Briscoe, Page, 2018, Verd, Crombach, Jaeger, 2017, Verd, Monk, Jaeger, 2018, Goodfellow, Phillips, Manning, Galla, Papalopulu, 2014). Here I investigate the conditions necessary for oscillations in these multiple feedback loop systems. The study is limited to ordinary differential equation models.

The general theory for predicting the onset of oscillations is presented in Section 2. Section 3 concerns the case of networks consisting of a loop and a sub-loop and explores optimal conditions for oscillations. Section 3.1 concerns the example of a single auto-repressive gene, and the species modelled are its mRNA concentration and the concentrations of its protein in the form of a monomer and of a dimer, see Fig. 1b. Section 3.2 concerns three-species systems with a loop and sub-loop, such as the ACDC motif, see Fig. 1c. Section 3.3 reverts to the *n*-species case and the optimal conditions for oscillations. Section 4 concerns networks with two interconnected rings, exploring a four-species example, which cannot be described as a loop with a sub-loop. Finally the results are discussed in Section 5.

## 2 The feedback system

In the most general case of a feedback system, there are *n* species and any species can regulate any other. This can be represented by a directed graph, with two types of edges. Sharp arrows indicate that the biochemical species at the base of the arrow positively influences the other, while flat-headed arrows indicate that the biochemical species at the base negatively influences the other. In gene regulatory networks, transcription of a gene is typically regulated by the protein of the other gene. Proteins that do this are referred to as transcription factors. Therefore, to a good approximation, it is the mRNA of the second gene that is directly regulated by the protein of the first. In some models, this is dealt with directly (see for example Elowitz, Leibler, 2000, Page, Perez Carrasco, 2018). It is common, however, to assume that the dynamics of mRNA are fast compared to those of protein so that their concentrations are at quasi- steady state. Here we not only consider transcriptional regulation, but also consider reactions between biochemical species, for example dimerisation of a protein.

The time evolution of the concentrations of the biochemical species of interest can be described by differential equations. If it is assumed that the biochemicals are well-mixed within the cell and sufficiently abundant, then ordinary differential equations can be employed (see Discussion for a description of more refined models in which this assumption is not made). Here we assume, in addition, that all species, be they mRNAs or proteins, degrade at a rate which is linear in their concentration. Within the general theory, it would be fairly straightforward to replace this assumption with degradation at a rate which is monotonically increasing in concentration (see for example Alrikaby, 2018).

As in Page and Perez Carrasco (2018), we model the concentrations of the species with a system of ordinary differential equations:

(1) $$\dot{x_{i}}=d_{i}\left(f_{i}\left(\underline{x}\right)-x_{i}\right),$$

where *d_i_* is the degradation rate of species *i* and *d_i_f_i_* describes its regulation by the other species. *f_i_* is some function of the concentration of the *n*-species. In the case of a ring oscillator, *f_i_* depends only on $x_{(i-1)\;\mathrm{mod}\;n}$ (see Fig. 1a). In addition, the number of down-regulations is odd.

The steady states of the system 1 are given by solutions to the system of equations:

$$\underline{f}\left(\underline{x}\right)=\underline{x}.$$

A straightforward argument shows that, for the ring oscillator, there is a unique steady state (see for example Page and Perez Carrasco, 2018). Rings in which there are an even number of down-regulations typically show multi-stability and are more complicated to analyse (see e.g. Smith, 1987, Müller, Hofbauer, Endler, Flamm, Widder, Schuster, 2006, Strelkowa, Barahona, 2010). We assume that since we model chemical reactions, transcription and translation only, the dynamics will be bounded. We look for the onset of oscillations by locating Hopf bifurcations. According to linear analysis, these occur when the eigenvalues of the Jacobian matrix at the steady state are imaginary.

We consider a Hopf bifurcation in such a system and derive the characteristic equation, setting the eigenvalue to be *iα*:

$$\begin{array}{ccc} 0 & = & \det\left(\begin{array}{cccc} -i\alpha -d_{1}+d_{1}\frac{\partial f_{1}}{\partial x_{1}} & d_{1}\frac{\partial f_{1}}{\partial x_{2}} & \cdots & d_{1}\frac{\partial f_{1}}{\partial x_{n}}\\ d_{2}\frac{\partial f_{2}}{\partial x_{1}} & -i\alpha -d_{2}+d_{2}\frac{\partial f_{2}}{\partial x_{2}} & \cdots & d_{2}\frac{\partial f_{2}}{\partial x_{n}}\\ \vdots & \vdots & \vdots & \vdots \\ \delta_{n}\frac{\partial f_{n}}{\partial x_{1}} & \cdots & d_{n}\frac{\partial f_{n}}{\partial x_{n-1}} & -i\alpha -d_{n}+d_{n}\frac{\partial f_{n}}{\partial x_{n}} \end{array}\right)\\ & = & \left(\prod_{k=1}^{n}d_{k}\right)\sum_{\sigma \in S_{n}}sgn\left(\sigma \right)\prod_{j=1}^{n}\left(\frac{\partial f_{j}}{\partial x_{\sigma_{j}}}-\left(1+i\alpha /d_{j}\right)\delta_{j\sigma_{j}}\right), \end{array}$$

where all the partial derivatives are taken at the relevant steady state and *δ* is the Kronecker delta. For a given permutation *σ*, let us divide the elements of {1,2,⋯,n} into those for which $j=\sigma_{j},$ which we call fixed points, and those for which this is not true, which we call moving points. The contribution from the term corresponding to *σ* in the determinant is only nonzero if the moving points of *σ* correspond to a set of mutually exclusive feedback loops. It is straightforward to see this: *σ* can be decomposed into a product of cycles and the term in the determinant corresponding to *σ* is a product of the products over these cycles. For the moving points, the product for each cycle is simply the products of the derivative of *f_j_* with respect to $x_{\sigma_{j}}$ within the cycle. These are only nonzero if species *σ_j_* regulates species *j* for each *j* in the cycle and therefore the cycle forms a feedback loop. Therefore, further assuming that there is not direct self-regulation of species ($\frac{\partial f_{i}}{\partial x_{i}}=0$ for all *i*), the characteristic equation is given by:

(2) $$0=\sum_{\begin{array}{c} S\in \;\text{set}\;\text{of}\;\text{sets}\;\text{of}\;\text{mutually}\\ \text{exclusive}\;\text{feedback}\;\text{loops}\; \end{array}}{\left(-1\right)}^{l_{S}}H_{S}\prod_{j\notin (\cup_{X\in S}X)}\left(1+i\alpha /d_{j}\right),$$

where *l_S_* is the number of loops in *S* and *H_S_* is the product of the gradients of the functions representing each link in each loop in *S* at the steady state. Hence positive feedback loops give rise to positive parameters, *H_S_*, and negative feedback loops give rise to negative parameters (the overall sign of *H_S_* is the product of the signs of the individual loops). The parameter is multiplied by a factor $\left(1+i\alpha /d_{j}\right)$ for each value of *j* representing a species not in the set of feedback loops. For example, the original ring contains all species and is therefore not multiplied by any of these factors. We must also include the empty set in the sum, with $H_{\emptyset }=1$. Eq. (2) provides a systematic way of organising the characteristic equation of an ODE model of a regulatory network. It will facilitate the identification of Hopf bifurcations.

## 3 Analysis of system with a loop and a sub-loop

Let us start by considering the case where there is a ring feedback involving all the species which is a negative feedback and in which there is one feedback loop other than the ring and the empty set, we refer to *H* for the whole ring as *F* and *H* for this other loop as *G*. We refer to the set of species involved in the other feedback loop as *W*. We define $\theta_{j}=\tan^{-1}\left(\alpha /d_{j}\right),$ for j=1,.,n, with *θ_j_* ∈ [0, *π*/2). Then the characteristic equation becomes

(3) $$F=\prod_{j\notin W}\left(1+i\tan\theta_{j}\right)\left[-G+\prod_{j\in W}\left(1+i\tan\theta_{j}\right)\right].$$

When *G* was zero, this had a solution for minimal |*F*| when $\sum_{j=1}^{n}\theta_{j}=\pi$. We can show that if *G* is greater than zero, the value of $\sum_{j=1}^{n}\theta_{j},$ and hence *α* and hence |*F*| at the bifurcation is increased, whereas if *G* is negative it is decreased.

Since in the case with just a ring, oscillations were favoured by equal degradation rates and since there is no means to distinguish within the characteristic equation between species involved in the same feedback loops, we assume that the degradation rates within *W* are the same and the degradations of the other species are the same as each other, but potentially different from those of the species in *W*. We call angles within *W, θ*, and those outside *W, ϕ*. Then

(4) $$F={\left(1+i\tan\phi \right)}^{n-|W|}\left[-G+{\left(1+i\tan\theta \right)}^{\left|W\right|}\right].$$

Therefore

(5) $$\left|F\right|=\sec^{n-|W|}\phi \left|-G+{\left(1+i\tan\theta \right)}^{\left|W\right|}\right|$$

and

(6) $$\pi =\left(n-|W|\right)\phi +Arg\left(-G+{\left(1+i\tan\theta \right)}^{\left|W\right|}\right).$$

Consider the sine rule applied to the triangle in the complex plane whose vertices are the origin and the points −G and $-G+{\left(1+i\tan\theta \right)}^{\left|W\right|}$. This gives

(7) $$\frac{G}{\sin[|W|\theta +(n-|W|)\phi ]}=\frac{\sec^{\left|W\right|}\theta }{\sin(n-|W|)\phi }=\frac{\left|-G+{\left(1+i\tan\theta \right)}^{\left|W\right|}\right|}{\sin|W|\theta }.$$

This means that

(8) $$\left|F\right|=\sec^{n-|W|}\phi \sin\left|W\right|\theta \frac{\sec^{\left|W\right|}\theta }{\sin(n-|W|)\phi }$$

and

(9) $$\tan\left(n-|W|\right)\phi =\frac{\sin|W|\theta }{G\cos^{\left|W\right|}\theta -\cos\left|W\right|\theta }.$$

Suppose, *G* is fixed and we want to minimise the value of |*F*| at the bifurcation. We first pause and study a couple of simple examples, with n=3 and |W|=2.

### 3.1 Simplest case incorporating a reversible reaction

Let us consider a loop with three species with one repression and two activations. One of the activations represents a dimerisation reaction, which is reversible, so there is also a reverse positive link from the dimer (A) to the monomer (C). Crucially this gives us a unique steady state. If the third species (B) is supposed to be the corresponding mRNA which is transcribed to form C, then this can be considered a model of negative auto-regulation of a gene.

The dynamical system is given by

(10) $$\begin{array}{ccc} \dot{a} & = & kc^{2}-la-d_{a}a\\ \dot{b} & = & d_{b}\left(f\left(a\right)-b\right)\\ \dot{c} & = & d_{c}\left(g\left(b\right)-c\right)+2la-2kc^{2}, \end{array}$$

where *f* is a decreasing function and *g* is an increasing function. Typically, models of gene regulation employ linear functions for *g*, assuming that protein is produced at a rate proportional to the level of mRNA. *k* denotes the forward reaction rate for the dimerisation reaction and *l* the reverse rate. *d_a_, d_b_* and *d_c_* are the degradation rates of dimer, mRNA (or whatever this species is) and monomer respectively. Let us denote the steady state of the system of Eq. (10) by (a_*_, b_*_, c_*_) and for the sake of brevity, denote *f*′(a_*_, b_*_, c_*_) by *f*′ and *g*′(a_*_, b_*_, c_*_) by *g*′.

The characteristic equation is given by

$$-2kc_{*}\left|f^{\prime }\right|g^{\prime }=\left(1+\lambda /d_{b}\right)[\left(d_{a}+\lambda +l\right)\left(1+\lambda /d_{c}+4kc_{*}/d_{c}\right)-4lkc_{*}/d_{c}].$$

At the Hopf bifurcations, λ=iα. Taking imaginary parts:

(11) $$\alpha^{2}=d_{a}d_{b}+d_{b}d_{c}+d_{c}d_{a}+l\left(d_{c}+d_{b}\right)+4kc_{*}\left(d_{a}+d_{b}\right).$$

Taking real parts gives:

(12) $$\begin{array}{ccc} 2kc_{*}f^{\prime }g^{\prime }/d_{a}= & & -\left[\left(1+4kc_{*}/d_{c}\right)/d_{a}+\left(1+l/d_{a}\right)/d_{c}\right]\\ & & \times \;[d_{b}+d_{a}+d_{c}+l+4kc_{*}+d_{c}d_{a}/d_{b}+ld_{c}/d_{b}\\ & & +\;4kc_{*}d_{a}/d_{b}]. \end{array}$$

Varying *d_b_*, the modulus of *f*′*g*′ is minimised when $d_{b}=\sqrt{d_{a}d_{c}+ld_{c}+4kc_{*}d_{a}}$.

Using this optimal value of *d_b_*, we get

$$\begin{array}{ccc} 2kc_{*}f^{\prime }g^{\prime }/d_{a}= & & -\left[\left(1+4kc_{*}/d_{c}\right)/d_{a}+\left(1+l/d_{a}\right)/d_{c}\right]\\ & & \times \left[d_{a}+d_{c}+l+4kc_{*}+2\sqrt{d_{a}d_{c}+ld_{c}+4kc_{*}d_{a}}\right]. \end{array}$$

This implies

$$\begin{array}{ccc} |f^{\prime }g^{\prime }| & = & \frac{1}{d_{c}2kc_{*}}\left(d_{c}+4kc_{*}+l+d_{a}\right)(d_{c}+4kc_{*}+l+d_{a}\\ & & +\;2\sqrt{d_{a}d_{c}ld_{c}+4kc_{*}d_{a}})\\ & = & \frac{2d_{c}}{4kc_{*}}\left(1+4kc_{*}/d_{c}+l/d_{c}+d_{a}/d_{c}\right)\\ & & \times \;(1+4kc_{*}/d_{c}+l/d_{c}+d_{a}/d_{c}\\ & & +\;2\sqrt{d_{a}/d_{c}+l/d_{c}+\left(4kc_{*}/d_{c}\right)d_{a}/d_{c}}). \end{array}$$

We can see that this is minimised when *l*/*d_c_* and *d_a_*/*d_c_* are as small as possible. In the limit as they tend to zero, we get

$$|f^{\prime }g^{\prime }|=\frac{2d_{c}}{4kc_{*}}{\left(1+4kc_{*}/d_{c}\right)}^{2},$$

which is minimised, at value 8, when $d_{c}=4kc_{*}$.

Therefore to optimise the chance of oscillations we want the dimer to be as stable and long lived as possible, whilst the rate at which monomers produce the dimer should be much larger than the dimeric degradation rate, but fourfold smaller than the monomeric degradation rate. At steady state, $kc_{*}^{2}=\left(l+d_{a}\right)a_{*}$. In order that $l+d_{a}<<4kc_{*},$ we require that c_*_ <  < a_*_, so at steady state, the majority of the molecules should exist in the dimerised state.

Using $d_{c}=4kc_{*}$ in the optimal value for *d_b_*, we get $d_{b}=\sqrt{\left(2d_{a}+l\right)d_{c}}$. This means that the fastest degradation rate is of the monomer, followed by the other species (which might be the corresponding mRNA), followed by the dimer. The frequency of the oscillations at the bifurcation will be $\sqrt{2d_{b}d_{c}}$.

We illustrate the results with numerical simulations for an example system. For the sake of simplicity, we consider that *g* is linear and *f* is piecewise linear. The function *f* takes value *f*_max_ for *a* < 0.9, it takes value 0 for *a* > 1.1 and between these values it is linear, decreasing from *f*_max_ to 0. This means that *f*′(*a*) only takes two values: 0 or $-5*f_{\text{max}}$. g(b)=mb, so that the production rate of the monomer is proportional to the level of species *B*, which might, for example represent the corresponding mRNA, whereby this is a model of auto-repressive gene regulation.

We set $l=d_{a}=0.1,$ $d_{b}=\sqrt{3}$. If $d_{c}=4kc_{*}=10,$ this gives ideal conditions for oscillations, which will occur approximately if |*f*′*g*′| > 8. If we set k=25, this is approximately true. As *d_c_* diverges from 4kc_*_, oscillations become progressively less likely. We therefore fix parameters as mentioned, set $f_{\text{max}}=1.0,$ m=5.0 and vary *d_c_*.

**Fig. 2:**
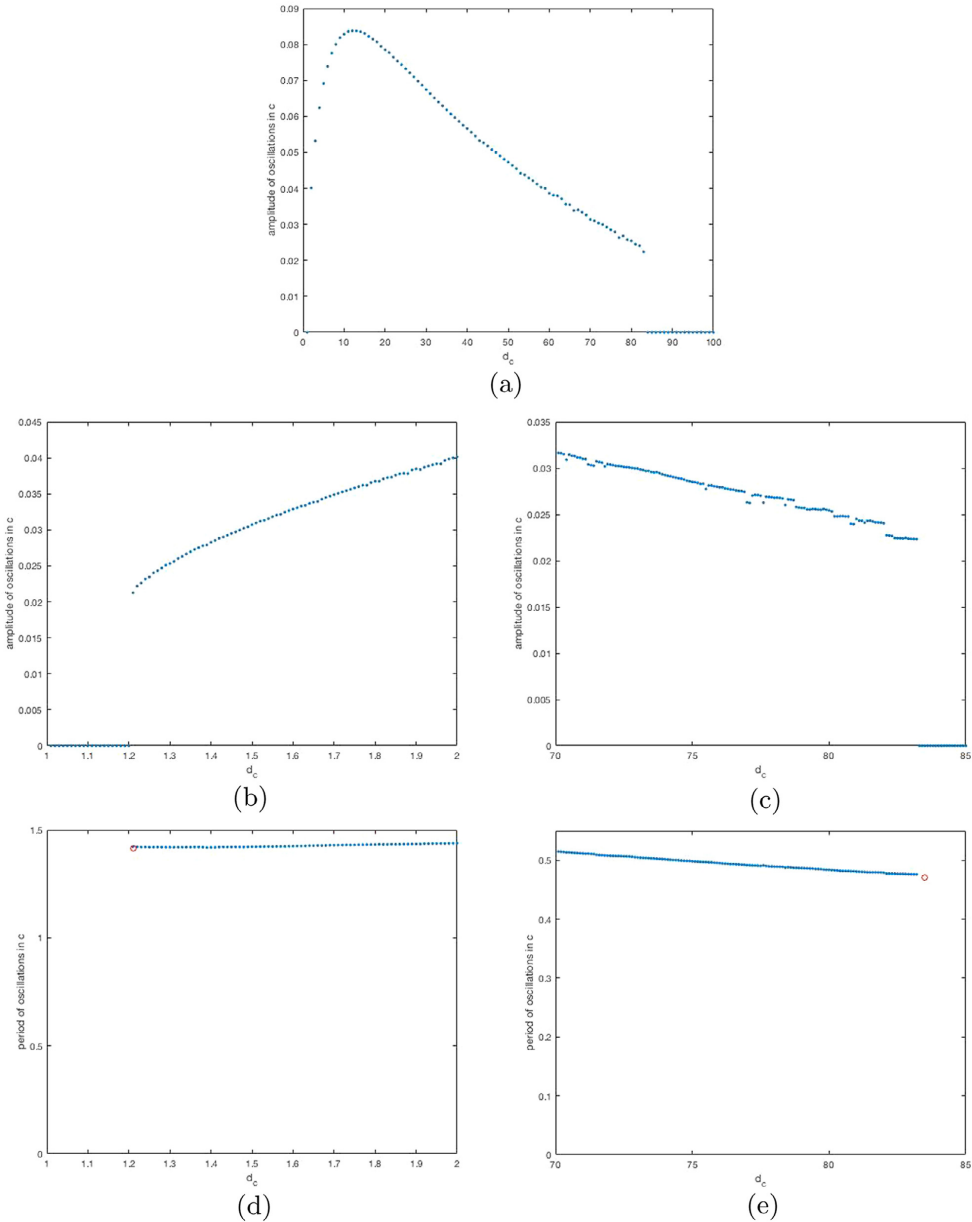
Bifurcation diagram with the amplitude of oscillations in species *C*, the monomer, (which we compute, approximately, as the maximum value of *c* minus its minimum value in the time interval [500,1000]) against the value of *d_c_*. Other parameters are given in the main text. We solve the ODEs using the Matlab ode solver ode23s. We use initial conditions, a=1.0,b=0.1 and c=0.1. In (a), we show the bifurcation diagram generated using values of $d_{c}=1.0,2.0,\cdots ,100.0$. In (b), we zoom in on the first bifurcation, using values $d_{c}=1.01,1.02,\cdots ,2.00$. In (c), we zoom in on the second bifurcation, using values $d_{c}=70.1,70.2,\cdots ,85.0$. In (d), we show the period of the oscillations against *d_c_* near the first bifurcation. In (e), we show the period of the oscillations against *d_c_* near the second bifurcation. We compute the period by identifying timepoints, in [500,1000], at which the concentration of monomer is less than its mean concentration (over [500,1000]), but at the next timepoint that is no longer true. We then divide the time difference between the first and last such timepoints by the number of such timepoints. In (d)-(e) we mark with a red circle the value of *d_c_* at the bifurcation and the period of oscillations there, as predicted by linear analysis. (For interpretation of the references to colour in this figure legend, the reader is referred to the web version of this article.)

In Fig. 2, we show numerically that indeed for intermediate values of *d_c_* there are oscillations. There are no oscillations when *d_c_* is too small or too large. It appears that there are two Hopf bifurcations at *d_c_* ≈ 1.25 and *d_c_* ≈ 82. Whether these are sub- or super-critical warrants further investigation (see Discussion).

Including a reversible reaction substantially changes the conditions for oscillations to occur. As before, they occur when the regulation functions are sufficiently steep at the steady state, but this time they are most likely to occur when the degradation rates differ between species.

Couching this in terms of the general system, we can no longer assume that the angles in the loop are the same, since one of the gradients is 2kc_*_ (see Discussion). Thus we must take the slightly more general form with three angles *θ_a_, θ_b_* and *θ_c_*, where $\tan\theta_{a}=\frac{\alpha }{l+d_{a}},$ $\tan\theta_{b}=\frac{\alpha }{d_{b}}$ and $\tan\theta_{c}=\frac{\alpha }{d_{c}+4kc_{*}}$. We get

$$\left|F\right|=\sec\theta_{b}\sin\left(\theta_{a}+\theta_{c}\right)\frac{\sec\theta_{a}\sec\theta_{c}}{\sin\theta_{b}}=\frac{\tan\theta_{a}+\tan\theta_{c}}{\tan\theta_{b}}\left(1+\tan^{2}\theta_{b}\right)=d_{b}\left[\frac{1}{l+d_{a}}+\frac{1}{d_{c}+4kc_{*}}\right]\left(1+\tan^{2}\theta_{b}\right)$$

and

$$\tan\theta_{b}=\frac{\sin(\theta_{a}+\theta_{c})}{G\cos\theta_{a}\cos\theta_{c}-\cos\left(\theta_{a}+\theta_{c}\right)},$$

where $G=4kc_{*}l/\left[\left(d_{a}+l\right)\left(d_{c}+4kc_{*}\right)\right]$ and $\left|F\right|=2kc_{*}f^{\prime }g^{\prime }d_{c}/\left[\left(l+d_{a}\right)\left(d_{c}+4kc_{*}\right)\right]$.

The equation for tan *θ_b_* gives

$$G-1+\tan\theta_{a}\tan\theta_{c}=d_{b}\left[\frac{1}{l+d_{a}}+\frac{1}{d_{c}+4kc_{*}}\right].$$

This implies:

$$\begin{array}{ccc} \alpha^{2} & = & \left(d_{a}+l\right)\left(d_{c}+4kc_{*}\right)[\frac{d_{b}}{d_{a}+l}+\frac{d_{b}}{d_{c}+4kc_{*}}+1\\ & & -\;\frac{4kc_{*}l}{\left(d_{a}+l\right)\left(d_{c}+4kc_{*}\right)}], \end{array}$$

which implies

$$\tan^{2}\theta_{b}=\frac{l+d_{a}}{d_{b}}+\frac{4kc_{*}+d_{c}}{d_{b}}+\frac{d_{a}d_{c}+ld_{c}+4kc_{*}d_{a}}{d_{b}^{2}}.$$

Therefore

$$\begin{array}{ccc} \left|F\right| & = & \left[\frac{1}{l+d_{a}}+\frac{1}{d_{c}+4kc_{*}}\right][d_{b}+l+d_{a}+4kc_{*}+d_{c}\\ & & +\;\frac{d_{a}d_{c}+ld_{c}+4kc_{*}d_{a}}{d_{b}}], \end{array}$$

which is the same result as above (Eq. (12)).

If we have two species involved in a negative feedback loop, e.g. when the protein corresponding to a gene represses the gene’s transcription, then in the simplest well-mixed model, there can be no oscillations, since there are only two species. If, however, the protein dimerises before acting as a repressor, there can be oscillations, since the number of species is now three. Two-species systems can show oscillations in models in which there are delays, see Monk (2003), Jensen et al. (2003), Lewis (2003), and Momiji and Monk (2008). It is possible to think of the two-species delay system as a higher dimensional system without delays. Delays may be due to the time taken for transcription, for example. In that case, it would alternatively be possible to model the levels of transcripts of different lengths and not explicitly use delays. Similarly, oscillations are possible in models in which there are two chemical species and diffusion (Glass, Kauffman, 1972, Shymko, Glass, 1974, Sturrock, Terry, Xirodimas, Thompson, Chaplain, 2011, Sturrock, Terry, Xirodimas, Thompson, Chaplain, 2012, Sturrock, Hellander, Matzavinos, Chaplain, 2013). However, in the papers by Sturrock and co-workers, the number of mathematical species is at least four, since the protein and mRNA have a nuclear and a cytoplasmic pool. In Glass and Kauffman (1972) and Shymko and Glass (1974) a two-species system is given which exhibits oscillations when there is diffusion, but the authors demonstrate in the appendix of Glass and Kauffman (1972) that a simpler ordinary differential equation model with only two-compartments shows the same behaviour. We also note that if we explicitly model the state of the enhancer or promoter which controls the gene’s expression, then this introduces a third species into the model of the single auto-repressive gene. It is common to assume that binding and unbinding to the DNA is fast compared to the dynamics of mRNA and protein. Assuming the binding is at quasi-equilibrium reduces the dimension of the resulting system, so that in the well-mixed system without delays, oscillations are impossible. However if DNA binding and unbinding occur on a timescale similar to transcription, translation and decay of protein and mRNA, then the system is three-dimensional and oscillations are possible (François, Hakim, 2005, Morant, Thommen, Lemaire, Vandermoëre, Parent, Lefranc, 2009). Stochastic oscillations can even occur in such a three-dimensional systems when the regulation of transcription is linear, which would preclude oscillations in the deterministic system (Wang et al., 2014).

### 3.2 The ACDC network and other three-species systems with a loop and a sub-loop

The ACDC network is a simple gene circuit consisting of a negative feedback loop between three species, with one reverse link, making a positive feedback loop between two of the species. It is hence a superposition of a repressilator and a toggle switch (Panovska-Griffiths, Page, Briscoe, 2013, Perez Carrasco, Barnes, Schaerli, Isalan, Briscoe, Page, 2018). It can display either switches between expression of the three genes as an input parameter (e.g. a morphogen signal) is changed or oscillations. For some values of the parameters, a stable steady state and a stable limit cycle can coexist. When there are stochastic fluctuations, this can lead to spontaneous switching on and off of oscillations. It has been proposed that the gap gene system, which patterns the anterior-posterior axis in the Drosophila melanogaster embryo, consists of three linked ACDC circuits, two of which operate in the DC (switch-like) regime and one in the AC (oscillatory) regime (Verd, 2016). More complex networks showing multiple negative and positive feedback loops have been proposed to control circaidian rhythms in Drosophila (Leloup and Goldbeter, 1998).

This simplest case of a system with a loop and a sub-loop represents the ACDC network if *G* is positive.

(13) $$\tan\phi =\frac{\sin2\theta }{G\cos^{2}\theta -\cos2\theta }=\frac{2\tan\theta }{G-1+\tan^{2}\theta }.$$

Let t=tanθ and $\lambda =\frac{\tan\phi }{t}=\frac{d_{\text{in}}}{d_{\text{out}}},$ where *d*_in_ is the degradation rate of species in the sub-loop and *d*_out_ is the degradation rate of species not in the sub-loop. In the notation of Section 3.

(14) $$\lambda =\frac{2}{G-1+t^{2}}$$

and

(15) $$\left|F\right|=\sec\phi \sin2\theta \frac{\sec^{2}\theta }{\sin\phi }=2\frac{t}{\cos^{2}\phi \tan\phi }=2\frac{1}{\lambda }\left(1+\tan^{2}\phi \right)=\frac{2}{\lambda }+2\lambda t^{2}.$$

Now we have $G-1+t^{2}=\frac{2}{\lambda },$ so $t^{2}=1-G+\frac{2}{\lambda }$. Therefore

(16) $$\left|F\right|=\frac{2}{\lambda }+2\lambda \left[1-G+\frac{2}{\lambda }\right]=4+\frac{2}{\lambda }+2\left(1-G\right)\lambda .$$

This is minimised at $4[1+\sqrt{1-G}]$ when $\lambda =\frac{1}{\sqrt{1-G}},$ provided *G* < 1. This means that when *G* > 0, i.e. the second loop represents a positive feedback loop, *λ* > 1, so that the degradation of species in the second loop is faster than that of those outside the second loop. In the case, *G* > 1, we get λ<2/(G−1) and hence |F|>G−1. |*F*| is minimised at G−1 when $\lambda =\frac{2}{G-1}$. Therefore the optimal value of *λ* is  > 1 provided *G* < 3. Therefore with a positive feedback sub-loop, oscillations are favoured when the degradation of the species in the sub-loop is faster that of those outside the loop, provided the feedback loop is not too strong. If it is very strong then oscillations are favoured when the degradation rate of the species in the sub-loop is slower than those outside. The oscillations that will form when the ratio of the degradation rates is close to $\frac{2}{G-1},$ have very low frequency. When 0 < *G* < 1, the oscillations have an angular frequency which decreases as *G* increases from $\sqrt{3}d_{\mathrm{in}}$ (when G=0, optimally *d_in_* is the same as the degradation rate of the species outside the sub-loop).

By contrast, when *G* < 0, i.e. the sub-loop represents a negative feedback loop, *λ* < 1, so that the degradation of species in the sub-loop is slower than that of those outside the sub-loop. In terms of angular contribution to the oscillations, those species involved in coherent sub-loops (which have the same sign as the outer loop) contribute a greater angle than those outside the sub-loop, whereas those involved in incoherent loops have a lesser contribution than those outside the sub-loop. An exception is when there is a very strong incoherent loop. We note that for *G* < 0, the angular frequency of oscillations decreases when compared to the degradation rate of the species outside the sub-loop, until it tends to a value equal to that degradation rate. The degradation rate of the species within the sub-loop becomes much smaller, so the oscillations increase in frequency compared to it.

We note that in the case *G* < 0, the steady state remains unique. In the case *G* > 0, there may be more than one steady state.

The value of |*F*| at the bifurcation is minimised when *G* is slightly greater than 1. Thus, counterintuitively, the probability of oscillations is maximised when there is a positive feedback loop embedded in the negative feedback loop. The ACDC network gives rise to oscillations more readily than the repressilator, provided that *G* < 9. By contrast, when there is an embedded negative feedback loop, oscillations are less likely than in the repressilator. For 1 < *G* < 3, the probability of oscillations is greater than for 0 < *G* < 1.

**Fig. 3:**
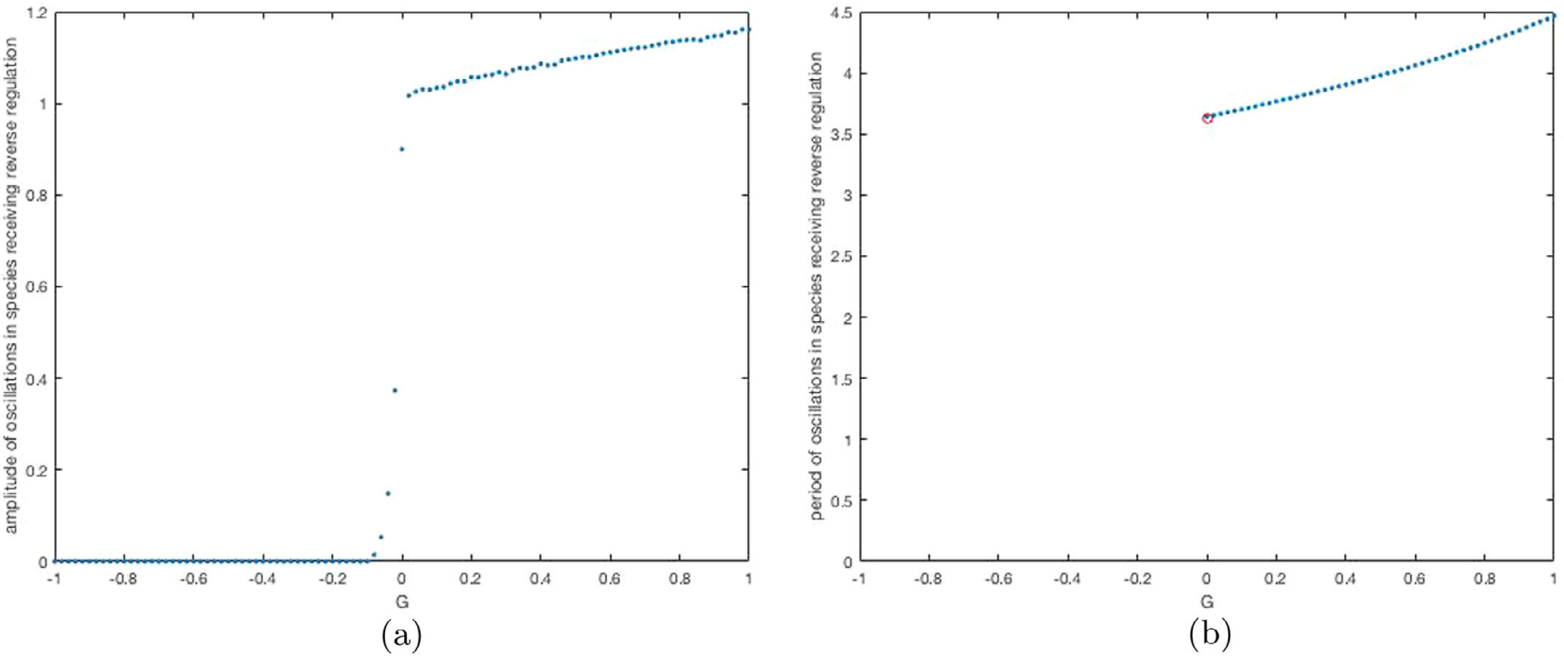
(a) Bifurcation diagram plotting the amplitude of oscillations in the species receiving the reverse regulation, in a gene regulatory network with a negative feedback ring and a sub-ring, against the sub-ring feedback coefficient *G*. We compute the amplitude, approximately, as the maximum concentration minus the minimum in the time interval [500,1000]. Other parameters are given in the main text. We solve the ODEs using the Matlab ode solver ode23s. We use initial conditions, (2.0,1.1,1.1), and plot the amplitude of oscillations in the species receiving the reverse regulatory link (species *A* in Fig. 1c which assumes *G* > 0, i.e. the link is repressive). In (b), we show the period of the oscillations against *G*. We compute this as in Fig. 2. We mark with a red circle the value of *G* at the bifurcation and the period of the oscillations there as predicted by linear analysis. (For interpretation of the references to colour in this figure legend, the reader is referred to the web version of this article.)

Note: We find that for all values of *G* < 0 used in Fig. 3, the oscillations in simulations are actually decaying, but when *G* is just below zero, the amplitude is still significant at t=500.

We illustrate this in Fig. 3 by showing the results of numerical simulations. We plot the maximal amplitude of oscillations (maximum concentration minus minimum concentration in the time window [500,1000] for a gene regulatory network with a negative feedback ring (with repression functions equal to 2 for *x* < 1/2, 3−2x for 1/2 < *x* < 3/2 and 0 for *x* > 3/2) and a second feedback loop (with a reverse repression or activation given by Max (1−k+kx,0), which multiplies the other input to that species), see Fig. 3a. This is the ACDC motif, if the reverse link is a repression (see Fig. 1c). Thus |F|=8 and G=−2k takes various values. We use the same initial condition, $x_{1}=2,$ $x_{2}=1.1$ and $x_{3}=1.1$. We see that sustained oscillations are possible for *G* > 0. We also plot the period of oscillations against *G* (Fig. 3b) and show for comparison the analytically derived values of *G* at the bifurcation and the analytically computed period 2*π*/*α*.

### 3.3 *n*-Species case

Returning to the general case. We again want to minimise the value of |*F*| at the bifurcation keeping *G* fixed.

(17) $$\begin{array}{ccc} \frac{d|F|}{d\theta } & = & \left|W∥F|(\cot|W|\theta +\tan\theta \right)\\ & & +\;\frac{d\phi }{d\theta }(n-|W|)|F|(\tan\phi -\cot(n-|W|)\phi ). \end{array}$$

Now

$$\begin{array}{ccc} & & \left(n-|W|\right)\sec^{2}\left(n-|W|\right)\phi \frac{d\phi }{d\theta }\\ & & \;=\left|W\right|\frac{\begin{array}{c} (G\cos^{\left|W\right|}\theta -\cos|W|\theta )\cos|W|\theta \\ \;-\sin|W|\theta (-G\cos^{\left|W\right|}\theta \tan\theta +\sin|W|\theta ) \end{array}}{(G\cos^{\left|W\right|}\theta -{\cos|W|\theta )}^{2}}\\ & & \;=\left|W\right|\frac{[G\cos^{|W|-1}\theta \cos(|W|-1)\theta -1]}{(G\cos^{\left|W\right|}\theta -{\cos|W|\theta )}^{2}}. \end{array}$$

So $\frac{d|F|}{d\theta }=0$ implies

(18) $$\begin{array}{ccc} \cot|W|\theta & + & \tan\theta =\left(\cot(n-|W|)\phi -\tan\phi \right)\cos^{2}\left(n-|W|\phi \right)\\ & & \times \;\frac{[G\cos^{|W|-1}\theta \cos(|W|-1)\theta -1]}{(G\cos^{\left|W\right|}\theta -{\cos|W|\theta )}^{2}}. \end{array}$$

Substituting for tan(n−|W|)ϕ, we get

$$\begin{array}{ccc} \tan\phi & = & \cot(n-|W|)\phi -(\cot|W|\theta +\tan\theta )\\ & & \frac{G\cos^{\left|W\right|}\theta (G\cos^{\left|W\right|}\theta -2\cos|W|\theta )+1}{G\cos^{|W|-1}\theta \cos\left(|W|-1\right)\theta -1}\\ & = & \frac{(G\cos^{\left|W\right|}\theta -\cos|W|\theta )(G\cos^{\left|W\right|}\theta \cos(|W|-1)\theta -\cos\theta )}{\sin|W|\theta [G\cos^{\left|W\right|}\theta \cos(|W|-1)\theta -\cos\theta ]}\\ & & -\;\frac{\cos(|W|-1)\theta (G\cos^{\left|W\right|}\theta (G\cos^{\left|W\right|}\theta -2\cos|W|\theta )+1)}{\sin|W|\theta [G\cos^{\left|W\right|}\theta \cos(|W|-1)\theta -\cos\theta ]}\\ & = & \frac{-\sin\theta -G\cos^{\left|W\right|}\theta \sin\left(|W|-1\right)\theta }{G\cos^{\left|W\right|}\theta \cos\left(|W|-1\right)\theta -\cos\theta }. \end{array}$$

We note that for small *G*, this means that tan *ϕ* is approximately tan *θ*, so that *θ* ≈ *ϕ*. Also from Eq. (8), tan(n−|W|)ϕ=−tan|W|θ, so tan(n−|W|)ϕ=−tan|W|ϕ, which implies nϕ=mπ, where *m* is an integer. We note that in this case $\left|F\right|=\sec^{n}\phi ,$ with *ϕ* ∈ [0, *π*/2), so |*F|* is minimal when *ϕ* takes the smallest possible value. This recapitulates previous results (Page and Perez Carrasco, 2018), giving θ=ϕ=π/n, as the approximately optimal condition for oscillations.

When *G* is large, in order for |*F*| to be reasonably small, we require from Eq. (4), $G\approx {\left(1+i\tan\theta \right)}^{\left|W\right|},$ so that sin |*W*|*θ* ≈ 0 and therefore |*W*|*θ* ≈ *rπ* and $\sec^{\left|W\right|}\theta {\left(-1\right)}^{r}\approx G$. This implies that |secθ| is very large, so that *θ* ≈ *π*/2, which means |W|=2r. This only works if ${\left(-1\right)}^{r}=sgnG$. If this is true, tiny changes in *θ* can lead to large changes in the argument of $-G+{\left(1+i\tan\theta \right)}^{\left|W\right|}$. Therefore the argument of ${\left(1+i\tan\phi \right)}^{n-|W|}$ is relatively unconstrained and, in order that |*F*| is as small as possible, secϕ should be minimised. Therefore *ϕ* should be small. Thus if *G* is large, and |*W*| is 2*r*, where ${\left(-1\right)}^{r}=sgnG,$ the degradation rates of species within the sub-loop should be small and those of species outside the sub-loop large. The system is thus approximately equivalent to the sub-loop only, with the species outside it in quasiequilibrium with those inside. We have said that for the species in the loop, *θ* should be close to *π*/2, which means that their degradation rates should be close to zero. This, however, is at the Hopf bifurcation. What will happen is that, when the sub-loop has a very large negative coefficient, the system will oscillate for most values of the degradation rates. When the degradation rates are very small, oscillations will cease. For information on what happens when there is a strong positive feedback loop, see Strelkowa and Barahona (2010).

## 4 Two interconnected rings

Many cases of two interconnected rings can be viewed as a large ring with a single sub-ring, which we have already studied. A simple example, which cannot, is obtained by extending the ACDC network, such that the reverse link added to the repressilator forms another repressilator with a fourth gene (see Fig. 1d). Although there is an outer ring (*ACDB* in the figure) and a sub-ring (*BC* in the figure), there are more sub-loops to consider, corresponding to the three-species repressilators. The equations can be written as:

(19) $$\begin{array}{ccc} \dot{a} & = & d_{a}\left(f\left(b\right)-a\right)\\ \dot{b} & = & d_{b}\left(g\left(c,d\right)-b\right)\\ \dot{c} & = & d_{c}\left(h\left(a,b\right)-c\right)\\ \dot{d} & = & d_{d}\left(j\left(c\right)-d\right), \end{array}$$

where all functions *f, g, h* and *j* are decreasing in their arguments.

We note that this system can have multiple steady states. In the example version that we use for numerical simulation, below, there are five steady states.

The characteristic equation at any of these steady states is given by

$$\begin{array}{ccc} & & \left(1+\frac{\lambda }{d_{a}}\right)\left(1+\frac{\lambda }{d_{b}}\right)\left(1+\frac{\lambda }{d_{c}}\right)\left(1+\frac{\lambda }{d_{d}}\right)\\ & & -\;h_{b}g_{c}\left(1+\frac{\lambda }{d_{a}}\right)\left(1+\frac{\lambda }{d_{d}}\right)\\ & & -\;h_{b}j^{\prime }g_{d}\left(1+\frac{\lambda }{d_{a}}\right)-f^{\prime }h_{a}g_{c}\left(1+\frac{\lambda }{d_{d}}\right)-f^{\prime }g_{d}h_{a}j^{\prime }=0, \end{array}$$

where all derivatives are evaluated at the relevant steady state. Therefore^1^

$$\begin{array}{ccc} & & \left(1+\frac{\lambda }{d_{a}}\right)\left(1+\frac{\lambda }{d_{b}}\right)\left(1+\frac{\lambda }{d_{c}}\right)\left(1+\frac{\lambda }{d_{d}}\right)\\ & & =\;h_{b}g_{c}\left(1+\frac{f^{\prime }h_{a}}{h_{b}}+\frac{\lambda }{d_{a}}\right)\left(1+\frac{j^{\prime }g_{d}}{g_{c}}+\frac{\lambda }{d_{d}}\right). \end{array}$$

We see that this is symmetric with respect to *d_b_* and *d_c_*, so we assume the optimal condition for oscillations has $d_{b}=d_{c}$.

At the Hopf bifurcation, λ=iα, for some real angular velocity *α*.

Without loss of generality, we set $d_{c}=d_{b}=1,$ and substituting in we get:

$$\begin{array}{ccc} & & {\left(1+i\alpha \right)}^{2}\left(1+i\alpha /d_{a}\right)\left(1+i\alpha /d_{d}\right)\\ & & =\;h_{b}g_{c}\left(1+\frac{f^{\prime }h_{a}}{h_{b}}+\frac{i\alpha }{d_{a}}\right)\left(1+\frac{j^{\prime }g_{d}}{g_{c}}+\frac{i\alpha }{d_{d}}\right). \end{array}$$

If we further assume that the two negative feedback loops have equal coefficients $(f^{\prime }h_{a}g_{c}=j^{\prime }g_{d}h_{b}),$ then species *a* and *d* are involved in the characteristic equation in symmetric ways, so we assume the probability of oscillations will be optimised if $d_{a}=d_{d}=\delta$. Setting $f^{\prime }h_{a}/h_{b}=-L=j^{\prime }g_{d}/g_{c},$ we therefore get:

$${\left[\left(1+i\alpha \right)\left(1+i\alpha /\delta \right)\right]}^{2}=h_{b}g_{c}{\left(1-L+i\alpha /\delta \right)}^{2}.$$

Therefore

$$\left(1+i\alpha \right)\left(1+i\alpha /\delta \right)=\pm \sqrt{h_{b}g_{c}}\left(1-L+i\alpha /\delta \right).$$

Taking the imaginary part gives and observing that the left hand side of the equation is positive gives:

$$1+1/\delta =\sqrt{h_{b}g_{c}}/\delta .$$

This means

$$h_{b}g_{c}={\left(\delta +1\right)}^{2}.$$

So, it should be easier to get oscillations, the smaller *δ* is. This means that in the model of two linked repressilators, oscillations are most likely when degradation of the species involved in the positive feedback loop is faster than that of the species not involved in the positive feedback loop. At the Hopf bifurcation, $\alpha =\sqrt{\delta }\sqrt{L+L\delta -\delta },$ which corresponds to an actual angular frequency of $\sqrt{d_{a}d_{b}}\sqrt{L+L\delta -\delta }$. If *δ* is very small, then the angular frequency of oscillations at the Hopf bifurcation will be approximately $\sqrt{L\delta }$ ($\sqrt{Ld_{a}d_{b}}$ in dimensional terms), which is intermediate between the degradation rates of the fast and the slow species.

**Fig. 4:**
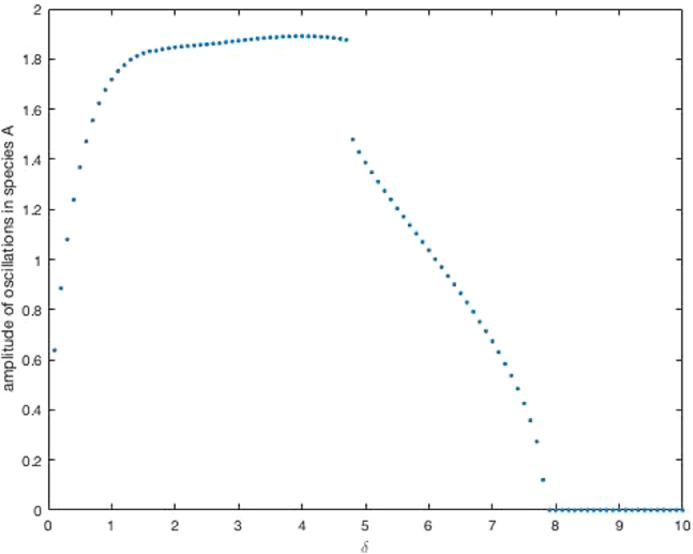
Bifurcation diagram plotting the amplitude of oscillations in species *A* against *δ* for numerical simulations of the system with two interconnected rings (Eqs. (19)). We compute the amplitude as in Fig. 2. We use δ=0.1,0.2,…10.0. Other parameters are as described in the main text. We solve the ODEs using the Matlab ode solver ode23s. We use initial conditions, a=1.4,b=1.0,c=1.7 and d=0.3.

In Fig. 4, we show numerically that in a simple example system with $f=3/\left(1+b^{4}\right),j=3/\left(1+c^{4}\right),g=10/\left(1+c^{4}+d^{4}\right)$ and $h=10/(1+a^{4}+b^{4}),$ there are oscillations when *δ* is small, but these cease when *δ* gets big enough.

This system exhibits multi-stability because of the existence of positive feedback loops as well as negative feedback loops. Therefore a more careful investigation of its bifurcations is warranted, see Discussion. In addition, the oscillations appear to occur around steady states which break the symmetry of the equations, i.e. the steady state values of *A* and *D* are different, as are those of *B* and *C*. It is therefore difficult to impose the symmetry condition that the repressilator coefficients are equal. A piecewise linear model could be employed in order to create a direct numerical comparison with the analytic results. This is beyond the scope of the current study. Nevertheless, simulations do show that for high *δ* oscillations are switched off. This is to be expected, since the system is then effectively a bistable switch between species *B* and *C*.

The structure of this four-species model is similar to a model of the p53-Mdm2 network response to DNA damage presented in Abou-Jaoudé et al. (2009) and Ouattara et al. (2010). The difference there is that the links from *C* to *D* and from *D* to *B* (Fig. 1d) are positive (see Fig. 1 of Abou-Jaoudé et al., 2009) and their direction is reversed relative to the model presented here.

## 5 Conclusions and discussion

In this paper, I have presented a systematic way (Eq. (2)) of organising the characteristic equation for the eigenvalues of the Jacobian matrix which determine the stability of steady states in regulatory networks. This facilitates the identification of Hopf bifurcations. I have used this to determine optimal conditions for oscillations in regulatory networks.

The chance of oscillations in a ring oscillator is maximised if the degradation rates of the species are equal (Page and Perez Carrasco, 2018). This is independent of all details of the negative feedback loop. When the system has more than one feedback loop the situation is more complicated. For example, when there are two loops, one of which is negative and contains all the species, then if the other loop is also negative, the optimal degradation rates of the species within it are smaller than those of the species outside it. If the second loop is a positive feedback loop, then, provided it is not too strong, the opposite is true and species outside the loop should degrade more slowly.

Counterintuitively, adding a positive feedback loop to a repressilator increases the probability of oscillations, provided that the loop is not too strong, whereas adding another negative feedback loop decreases the probability of oscillations. The fact that positive feedback loops could enhance oscillations in a repressilator was already known for specific kinetic functions, see for example (Ananthasubramaniam and Herzel, 2014).

In Section 3.1, it is shown that the optimal conditions for oscillations for the autoregulatory gene model have the majority of the protein in its dimerised form at the steady state. The dimeric degradation rate and the rate at which the dimer dissociates to form monomers should both be small. The degradation rate of the mRNA should optimally be intermediate and the monomeric protein degradation rate large. This is unlikely to be typical of autoregulatory genes, since mRNA degradation rates are typically larger than protein degradation rates (e.g. Hoffmann, Levchenko, Scott, Baltimore, 2002, Nelson, Ihekwaba, Elliott, Johnson, Gibney, Foreman, Nelson, See, Horton, Spiller, et al., 2004) and may suggest that oscillations are most likely when some process explicitly removes monomeric protein. Degradation rates of Hes1 protein and mRNA are however similar (e.g. Jensen, Sneppen, Tiana, 2003, Hirata, Yoshiura, Ohtsuka, Bessho, Harada, Yoshikawa, Kageyama, 2002). Optimally $d_{c}=4kc^{*},$ which means that, at the steady state, only one in every three monomers dimerises whilst the other two degrade (see Eq. (10)). The optimal mRNA degradation rate is then $\sqrt{\left(2d_{a}+l\right)d_{c}}$. A simple closed form expression in terms of the parameters of the model is given for the angular frequency of the oscillations close to the bifurcation (Eq. (11)). This simplifies under the optimal assumptions listed above to $\alpha =\sqrt{2d_{b}d_{c}}$. Thus, under these conditions, the period of oscillations is intermediate between the timescale of degradation of the mRNA and the protein monomer. If, instead of assuming optimal conditions for oscillations, we simply assume that the dimeric protein does not degrade directly, then the angular frequency at the bifurcation is given by $\alpha =\sqrt{d_{b}d_{c}+ld_{c}+d_{b}4kc^{*}}$. Again assuming the optimal condition for one in every three monomers to form a dimer, gives $\alpha =\sqrt{d_{c}\left(2d_{b}+l\right)}$ and assuming that the dimeric unbinding rate is small compared to the mRNA degradation rate yields the same angular frequency as before of $\alpha =\sqrt{2d_{b}d_{c}},$ without assuming that the mRNA is longer lived than the monomeric protein. If the dimer less stable, this should speed up oscillations. A dimeric model of autoregulation of Hes1 with protein and mRNA degradation rates of approximately 1/(25 mins.) would predict an oscillation period at the bifurcation of 111 mins., assuming one in every three monomeric proteins dimerises and the unbinding rate of dimers is very low. This is close to the actual period (see e.g. Yoshiura et al., 2007). Much more rapid oscillations would be possible if the dimer were less stable. See Momiji and Monk (2008) and Sturrock et al. (2014) for more detailed models incorporating nuclear and cytoplasmic components and transcriptional and translational time delays. Whilst the detailed features of sub-cellular location are no doubt important, an angular frequency of $\sqrt{2}$ times the geometric mean of the mRNA and protein degradation rates may serve as a useful estimate.

In Section 4, we predict that for two linked repressilators (or equivalent negative feedback loops), see Fig. 1d, of equal strength (i.e. feedback coefficient), conditions for oscillations are optimised if the species forming the two-species positive feedback loop (*B* and *C* in the figure) degrade faster than the other species. *A* and *D* should have equal degradation rates, as should *B* and *C*. If *B* and *C* degrade very fast compared to the other two species, then the angular frequency of oscillations at the bifurcation will be $\alpha =\sqrt{Ld_{a}d_{b}},$ where −L is the coefficient of the negative feedback loop divided by the coefficient of the two-species positive feedback loop. This warrants further investigation, see below.

The dynamics of systems with more than one feedback loop are more complicated than those with a single feedback loop. There can be multiple stable steady states (as is true for a ring with a positive feedback sub-loop). There can be the co-existence of a stable steady state and a stable limit cycle, for example for the simple ACDC circuit, Perez Carrasco et al. (2018). Finally there can be chaos, Zhang et al. (2012), which is not possible for the negative feedback ring systems, Page and Perez Carrasco (2018).

In this paper, we only consider ordinary differential equation models of regulatory networks. Much interesting work has shown how oscillations can arise when there are only two biological species, but when there are delays (Lewis, 2003, Monk, 2003, Jensen, Sneppen, Tiana, 2003, Momiji, Monk, 2008), multiple compartments (Glass and Kauffman, 1972) and/ or diffusion (Busenberg, Mahaffy, 1985, Shymko, Glass, 1974, Cangiani, Natalini, 2010, Sturrock, Terry, Xirodimas, Thompson, Chaplain, 2011, Sturrock, Terry, Xirodimas, Thompson, Chaplain, 2012, Sturrock, Hellander, Matzavinos, Chaplain, 2013). As mentioned in Section 3, these cases are equivalent or similar in nature to ordinary differential equation models with a higher number of mathematical species. Nevertheless, sub-cellular location of biochemical species is clearly an important factor in the regulation of gene expression (e.g. Hoffmann, Levchenko, Scott, Baltimore, 2002, Nelson, Ihekwaba, Elliott, Johnson, Gibney, Foreman, Nelson, See, Horton, Spiller, et al., 2004). The environment of the cells is also noisy and so stochastic effects are important. For examples of numerical algorithms to simulate stochastic gene regulation and the implications of intrinsic noise in negative feedback systems, see Hegland et al. (2007) and Zeron and Santillán (2010).

Further work could address the inclusion of nonlinear degradation or other nonlinear negative self-regulatory terms for the species. This would allow the auto-regulatory gene model to be couched in terms of the general theory of Section 2 and would also facilitate application to a wider range of real gene regulatory models. Characterising the Hopf bifurcations as supercritical or subcritical would also be interesting. This is somewhat laborious, however, even for the case of a ring oscillator (see Page and Perez Carrasco, 2018). A better numerical bifurcation analysis of the four-species model and similar models, such as that of Abou-Jaoudé et al. (2009) and Ouattara et al. (2010), would also be desirable. The multiple steady states render finding Hopf bifurcations more difficult in this model. Finally, future work could extend the results on optimal conditions for oscillations to systems with delays, diffusion and stochasticity.

## Footnotes

1 In terms of the general theory presented in Section 2, in addition to the term corresponding to the empty set, we have terms corresponding to the feedback loops BC, ACB, BCD and ACDB.

## References

[bib0001] Abou-Jaoudé W., Ouattara D.A., Kaufman M. (2009). From structure to dynamics: frequency tuning in the p53–mdm2 network: i. logical approach. J. Theor. Biol..

[bib0002] Alon U. (2007). Network motifs: theory and experimental approaches. Nat. Rev. Genet..

[bib0003] Alrikaby Z. (2018). Stability and Hopf bifurcation analysis of lac operon model with distributed delay and nonlinear degradation rate. Math. Med.Biol..

[bib0004] Ananthasubramaniam B., Herzel H. (2014). Positive feedback promotes oscillations in negative feedback loops. PLoS ONE.

[bib0005] Angeli D., Ferrell J.E., Sontag E.D. (2004). Detection of multistability, bifurcations, and hysteresis in a large class of biological positive-feedback systems. Proc. Natl. Acad. Sci..

[bib0006] Bar-Or R.L., Maya R., Segel L.A., Alon U., Levine A.J., Oren M. (2000). Generation of oscillations by the p53-mdm2 feedback loop: a theoretical and experimental study. Proc. Natl. Acad. Sci..

[bib0007] Buşe O., Kuznetsov A., Pérez R.A. (2009). Existence of limit cycles in the repressilator equations. Int. J. Bifurcat. Chaos.

[bib0008] Buşe O., Pérez R., Kuznetsov A. (2010). Dynamical properties of the repressilator model. Phys. Rev. E.

[bib0009] Busenberg S., Mahaffy J. (1985). Interaction of spatial diffusion and delays in models of genetic control by repression. J. Math. Biol..

[bib0010] Cangiani A., Natalini R. (2010). A spatial model of cellular molecular trafficking including active transport along microtubules. J. Theor. Biol..

[bib0011] Cinquin O., Demongeot J. (2002). Positive and negative feedback: striking a balance between necessary antagonists. J. Theor. Biol..

[bib0012] De Jong H. (2002). Modeling and simulation of genetic regulatory systems: a literature review. J. Comput. Biol..

[bib0013] Dequéant M.-L., Glynn E., Gaudenz K., Wahl M., Chen J., Mushegian A., Pourquié O. (2006). A complex oscillating network of signaling genes underlies the mouse segmentation clock. Science.

[bib0014] Elowitz M.B., Leibler S. (2000). A synthetic oscillatory network of transcriptional regulators.. Nature.

[bib0015] François P., Hakim V. (2005). Core genetic module: the mixed feedback loop. Phys. Rev. E.

[bib0016] Glass L., Kauffman S.A. (1972). Co-operative components, spatial localization and oscillatory cellular dynamics. J. Theor. Biol..

[bib0017] Goodfellow M., Phillips N.E., Manning C., Galla T., Papalopulu N. (2014). Microrna input into a neural ultradian oscillator controls emergence and timing of alternative cell states. Nat. Commun..

[bib0018] Hegland M., Burden C., Santoso L., MacNamara S., Booth H. (2007). A solver for the stochastic master equation applied to gene regulatory networks. J. Comput. Appl. Math..

[bib0019] Hirata H., Yoshiura S., Ohtsuka T., Bessho Y., Harada T., Yoshikawa K., Kageyama R. (2002). Oscillatory expression of the bhlh factor hes1 regulated by a negative feedback loop. Science.

[bib0020] Hoffmann A., Levchenko A., Scott M.L., Baltimore D. (2002). The i*κ*b-nf-*κ*b signaling module: temporal control and selective gene activation. Science.

[bib0021] Jensen M., Sneppen K., Tiana G. (2003). Sustained oscillations and time delays in gene expression of protein hes1. FEBS Lett..

[bib0022] Kaufman M., Soulé C. (2019). On the multistationarity of chemical reaction networks. J. Theor. Biol..

[bib0023] Leloup J.-C., Goldbeter A. (1998). A model for circadian rhythms in drosophila incorporating the formation of a complex between the per and tim proteins. J. Biol. Rhythms.

[bib0024] Lewis J. (2003). Autoinhibition with transcriptional delay: a simple mechanism for the zebrafish somitogenesis oscillator. Current Biology.

[bib0025] Michael D., Oren M. (2003). The p53–mdm2 module and the ubiquitin system. Semin. Cancer Biol..

[bib0026] Momiji H., Monk N.A. (2008). Dissecting the dynamics of the hes1 genetic oscillator. J. Theor. Biol..

[bib0027] Monk N.A. (2003). Oscillatory expression of hes1, p53, and nf-*κ*b driven by transcriptional time delays. Current Biol..

[bib0028] Morant P.-E., Thommen Q., Lemaire F., Vandermoëre C., Parent B., Lefranc M. (2009). Oscillations in the expression of a self-repressed gene induced by a slow transcriptional dynamics. Phys. Rev. Lett..

[bib0029] Müller S., Hofbauer J., Endler L., Flamm C., Widder S., Schuster P. (2006). A generalized model of the repressilator. J. Math. Biol..

[bib0030] Nelson D., Ihekwaba A., Elliott M., Johnson J., Gibney C., Foreman B., Nelson G., See V., Horton C., Spiller D. (2004). Oscillations in nf-*κ*b signaling control the dynamics of gene expression. Science.

[bib0031] Ouattara D.A., Abou-Jaoudé W., Kaufman M. (2010). From structure to dynamics: frequency tuning in the p53-mdm2 network. ii: differential and stochastic approaches. J. Theor. Biol..

[bib0032] Page K.M., Perez Carrasco R. (2018). Degradation rate uniformity determines success of oscillations in repressive feedback regulatory networks. J R Soc. Interface.

[bib0033] Panovska-Griffiths J., Page K.M., Briscoe J. (2013). A gene regulatory motif that generates oscillatory or multiway switch outputs. J R Soc. Interface.

[bib0034] Perez Carrasco R., Barnes C.P., Schaerli Y., Isalan M., Briscoe J., Page K.M. (2018). Combining a toggle switch and a repressilator within the ac-dc circuit generates distinct dynamical behaviors. Cell Syst..

[bib0035] Plahte E., Mestl T., Omholt S.W. (1995). Feedback loops, stability and multistationarity in dynamical systems. J. Biol. Syst..

[bib0036] Reddy A.B., Rey G. (2014). Metabolic and nontranscriptional circadian clocks: eukaryotes. Annu. Rev. Biochem..

[bib0037] Shymko R., Glass L. (1974). Spatial switching in chemical reactions with heterogeneous catalysis. J. Chem. Phys..

[bib0038] Smith H. (1987). Oscillations and multiple steady states in a cyclic gene model with repression. J. Math. Biol.

[bib0039] Soulé C. (2003). Graphic requirements for multistationarity. ComPlexUs.

[bib0040] Strelkowa N., Barahona M. (2010). Switchable genetic oscillator operating in quasi-stable mode. J. R. Soc. Interface.

[bib0041] Sturrock M., Hellander A., Aldakheel S., Petzold L., Chaplain M.A. (2014). The role of dimerisation and nuclear transport in the hes1 gene regulatory network. Bull. Math. Biol..

[bib0042] Sturrock M., Hellander A., Matzavinos A., Chaplain M.A. (2013). Spatial stochastic modelling of the hes1 gene regulatory network: intrinsic noise can explain heterogeneity in embryonic stem cell differentiation. J. R. Soc. Interface.

[bib0043] Sturrock M., Terry A.J., Xirodimas D.P., Thompson A.M., Chaplain M.A. (2011). Spatio-temporal modelling of the hes1 and p53-mdm2 intracellular signalling pathways. J. Theor. Biol..

[bib0044] Sturrock M., Terry A.J., Xirodimas D.P., Thompson A.M., Chaplain M.A. (2012). Influence of the nuclear membrane, active transport, and cell shape on the hes1 and p53–mdm2 pathways: insights from spatio-temporal modelling. Bull. Math. Biol..

[bib0045] Verd B. (2016). EvoDevo in Phase Space: The Dynamics of gap gene Expression.

[bib0046] Verd B., Crombach A., Jaeger J. (2017). Dynamic maternal gradients control timing and shift-rates for drosophila gap gene expression. PLoS Comput. Biol..

[bib0047] Verd B., Monk N.A., Jaeger J. (2018). Modularity, criticality and evolvability of a developmental gene regulatory network. bioRxiv.

[bib0048] Wang J., Lefranc M., Thommen Q. (2014). Stochastic oscillations induced by intrinsic fluctuations in a self-repressing gene. Biophys. J..

[bib0049] Yoshiura S., Ohtsuka T., Takenaka Y., Nagahara H., Yoshikawa K., Kageyama R. (2007). Ultradian oscillations of stat, smad, and hes1 expression in response to serum. Proc. Natl. Acad. Sci..

[bib0050] Zeron E.S., Santillán M. (2010). Distributions for negative-feedback-regulated stochastic gene expression: dimension reduction and numerical solution of the chemical master equation. J. Theor. Biol..

[bib0051] Zhang Z., Ye W., Qian Y., Zheng Z., Huang X., Hu G. (2012). Chaotic motifs in gene regulatory networks. PLoS ONE.

